# Inner Peace needs of male psychiatric patients in post-war Croatia are associated with their needs to clarify open issues in their life and their needs for forgiveness

**DOI:** 10.3389/fpubh.2023.1095835

**Published:** 2023-09-18

**Authors:** Andrijana Glavas, Arndt Büssing, Klaus Baumann

**Affiliations:** ^1^Caritas Science and Christian Social Work, Faculty of Theology, Albert-Ludwig-University, Freiburg im Breisgau, Germany; ^2^Professorship Quality of Life, Spirituality and Coping, Faculty of Health, Witten/Herdecke University, Herdecke, Germany

**Keywords:** spiritual needs, nature, Inner Peace, forgiveness, post-war society, war participation, PTSD, Croatia

## Abstract

**Background:**

More than 25 years after the end of the Balkan war, many people belonging to the post-war population are still traumatized by the war events and have been treated for post-traumatic stress disorder or other psychiatric diagnoses. We were interested in their Inner Peace needs, how these relate to indicators of mental health, and their needs to clarify open processes in their lives and to forgive and be forgiven.

**Materials and methods:**

In a cross-sectional survey with standardized questionnaires (i.e., SpNQ, PCL-M, HADS, and BMLSS), 638 male patients who were treated in seven psychiatric centers in Croatia were enrolled. 68% were diagnosed with PTSD and 32% had other psychiatric diagnoses. Most had actively participated in the Balkan war (79%), and 60% for the whole war period.

**Results:**

Strong needs to “immerse into beauty of nature” were stated by 47%, to “dwell at a place of quietness and peace” by 66%, and to “find inner peace” by 57%. These Inner Peace needs were highest in men treated with PTSD diagnoses as compared to men with other psychiatric diagnoses and were slightly lower in men who were active during the whole war period as compared to shorter phases of war participation. Regression analyses with Inner Peace needs as a dependent variable revealed that Clarification/Forgiveness needs were the best predictor, with further influences of PTSD symptoms and life satisfaction, explaining altogether 49% of the variance. The best predictors of their PTSD symptoms were life satisfaction, perceived burden, depressive symptoms, Inner Peace needs, religious trust, and duration of war participation, explaining 60% of the variance.

**Conclusion:**

In Croatian male war participants in clinical treatment decades after the war, Inner Peace needs indicate their ongoing intention to let go of their disturbing experiences and to find states of inner peace, particularly at specific places of quietness and peace. These needs can be considered metaphors for longing for wholeness, integrity, and safety, in contrast to the ongoing impact of unresolved issues. Thus, apart from psychotherapeutic treatment, sheltered places of nature, inspiration, and reconciliation might be elements to improve the difficult situation of post-war victims still suffering from their experiences.

## 1. Introduction

The abominable war of Russian President Putin's army against Ukraine has triggered painful war memories in the populations involved in the Balkan war of the 1990s, including many Croatian veterans. When the war in Croatia (1991–1995) started, there was no Croatian army yet to defend the young state. Most of the Croatian population immediately realized they had to defend their country and autonomy by themselves. There was an overwhelming readiness for recruitment and active participation in the defense of their country and its people against Serbian aggression (1). Apart from soldiers who deserted from the Yugoslavian army, they were members of civil protection organizations or Croatian volunteers without any formation under arms or any military training. From one day to the other, they were fighting under arms and exposed to multiple violently traumatic events. Survivors experienced and witnessed explosions and destructions of buildings, violence and killings, displacements, imprisonments, torture, injuries, mutilations, and deaths—among their companions and their beloved ones, families, and friends. These events deeply imprinted themselves into the affective memories, frequently causing trauma and subsequent post-traumatic stress disorder (PTSD) in many of the combatants, their relatives, and any civil survivors of the war (2, 3).

According to official estimates of the Croatian government, at least one million people were exposed to the distress of the war, and approximately 7,600 Croatians—civil and active war participants—were detained in war prisons (4). At present, there are still a high number of people with symptoms of PTSD who feel persecuted by painful thoughts and memories (5). In everyday life, they try to avoid situations and activities that could remind them of their traumatic experiences. Nevertheless, they are frequently very irritable and prone to self-destructive behavior; they may feel overstrained and unable to cope, helpless and depressed, and inclined to abuse alcohol and other drugs, resulting in increased disease, suicidality, and mortality rates. In 80% of Croatian PTSD patients, there is a diagnosed comorbidity of depression (most frequently), anxiety disorder, addictive disorder, or other disorders (6). Kovačić Petrović et al. (7) reported that 78% of them had at least one (additional) somatic disorder that affected their general quality of life. A similar situation is found in veteran PTSD patients in Bosnia and Herzegovina, where 45% presented with chronic somatic diseases (8). One-third of Croatian veteran PTSD patients presented with some stress-related coronary disease (9).

The effects of experienced trauma were not only limited to the development of mental and physical illnesses, which meant a higher mortality risk for people with war experience (10) but also left deep emotional wounds, which often manifested themselves in dysfunctional perceptions of life by themselves and others. Since the number of PTSD patients in Croatia is still high, it can be assumed that many developed CPTSD (complex PTSD) over time. Unfortunately, no study has been conducted so far in Croatia at the national level that would have investigated the frequency of CPTSD patients according to the new ICD-11 (International Classification of Diseases, 11th revision) criteria.

The results of a smaller study (11) on war-traumatized patients treated for PTSD (*n* = 160) showed that the vast majority of them (80%) in fact met the criteria of PTSD. Patients suffering from CPTSD suffer from hopelessness, despair, and a frequently persistent depressed mood, which may be accompanied by latent chronic suicidality and severe self-injury (12). War veterans' low quality of life was shown to be influenced by personality-related factors such as harm avoidance and novelty seeking, as well as by self-directedness and cooperativeness (13). In addition, patients experience serious problems in interpersonal relationships and feelings of being inferior and worthless, often accompanied by feelings of guilt and shame (14). Patients with PTSD feel burdened by their condition; they face socioeconomic difficulties and victimization of their condition (15). There is a considerable correlation between greater war traumatization and lower quality of life. Croatian war veterans with PTSD have had a significantly poorer quality of life compared to veterans without PTSD (7, 16).

The consequences of these unresolved and stressful situations have been endured so many years after the war has ended, not only by the affected persons but also by their families and post-war generations.

The overcoming of traumatic experiences is a difficult and protracted process in which traumatized people have—no less than other patients—a need for a professionally competent and, at the same time, holistic perception of their person. This also includes their religious or spiritual orientations, struggles, and resources, which have been affected by the traumatic dynamics, too, and which, at the same time, could help these patients as resources to cope with their difficult situation. A few studies confirm the beneficial associations between religiosity and stress, psychological stability, and a better processing of the illness in the post-war period for patients with war-related PTSD (17).

Furthermore, veterans from other war zones, such as the Gulf War, Iraq War, and Afghanistan deployments, experienced PTSD symptoms (18, 19). In these veterans, post-trauma support had a positive influence on their wellbeing outcomes. A meta-analysis revealed that social support was a protective factor against PTSD, while low family support and low unit cohesion were triggers of PTSD symptoms (19). A psychiatric comorbidity may aggravate PTSD; however, in Afghanistan/Iraq era veterans, comorbid diagnoses did not worsen PTSD, “indicating that psychiatric comorbidity may be less important in considering the role of social support in PTSD” (20). More relevant as a PTSD predictor in war veterans might be psychological inflexibility, which is not overlapping with personality factors, esp. neuroticism, as found by Meyer et al. (21).

There are various other buffering factors, among them the ability to forgive. Veterans who were exposed to combat experience and suffer from PTSD are confronted with negative feelings such as anger, hostility, and aggression. These could be buffered when they are able to forgive. In a study among Turkish veterans, anger and negative affect “fully mediated the relationship between forgiveness and both PTSD and depression comorbid to PTSD” (22). In terror victims, decreased PTSD symptoms were related to forgiveness and problem-focused coping, while these symptoms were higher when emotion-focused strategies were used (23). Furthermore, people recovering from addiction show signs of PTSD, have feelings of guilt, and have difficulties forgiving themselves (24). These feelings of guilt predicted PTSD symptoms, while self-forgiveness did not predict the outcome (24). In contrast, in people with PTSD after traumatic accidents, forgiveness toward others did change their PTSD symptoms (25). These findings underline that the ability of traumatized people to forgive others may improve their PTSD burden. However, the context is relevant (26), and in some cases, forgiveness seems not to be possible (27). Nevertheless, greater religiosity seems to be a facilitator of forgiveness, while hostility would decrease it (28). Addressing the role of religious views on suicidal ideation among Iraq/Afghanistan-era veterans revealed that, after controlling for age, PTSD diagnosis, and substance use problems, their suicide attempts were interpreted as a punishment of God and a lack of meaning and purpose in life on the one hand and a lack of control and low ability of self-forgiveness on the other hand (29). This means both extrinsic and intrinsic resources are required to support post-war veterans. However, talking with military service members and post-war veterans about their experiences and mental trauma is problematic (30, 31). Therefore, an assessment of unmet psychosocial, existential, and spiritual needs is important as these may indicate a burden that is usually not communicated for fear of stigma (32).

In German soldiers who have experienced combat trauma, their stress perception and PTSD symptoms were moderately associated with existential and inner peace needs (32). The strongest needs were to “plunge into the beauty of nature”, to “dwell at a place of quietness and peace,” and to “find inner peace”. When these needs were high, their life satisfaction was reduced (and vice versa). In this context, the experience of nature can be regarded as a resource. In fact, experiences in nature can have positive effects on wellbeing and mood states (33, 34) and on stress perception (35). Furthermore, the systematic review by de Kaijzer et al. (36) stated that mental health and wellbeing can be improved by interaction with the natural environment.

A further finding from these soldiers was that 30% had a strong need to talk with others about their fears and worries, 13% had a strong need to forgive, and 13% had a strong need to be forgiven (37). Those who were treated in the hospital for psycho-mental trauma had significantly higher needs to clarify open aspects of their lives, to talk about their fears and worries, and to forgive and to be forgiven (37). Thus, the topics of inner peace needs (particularly peaceful places to distance oneself from the trauma and may experience ease and peacefulness) and needs to forgive and to be forgiven are associated, and both need categories are related to PTSD symptoms in soldiers.

**Figure 1:**
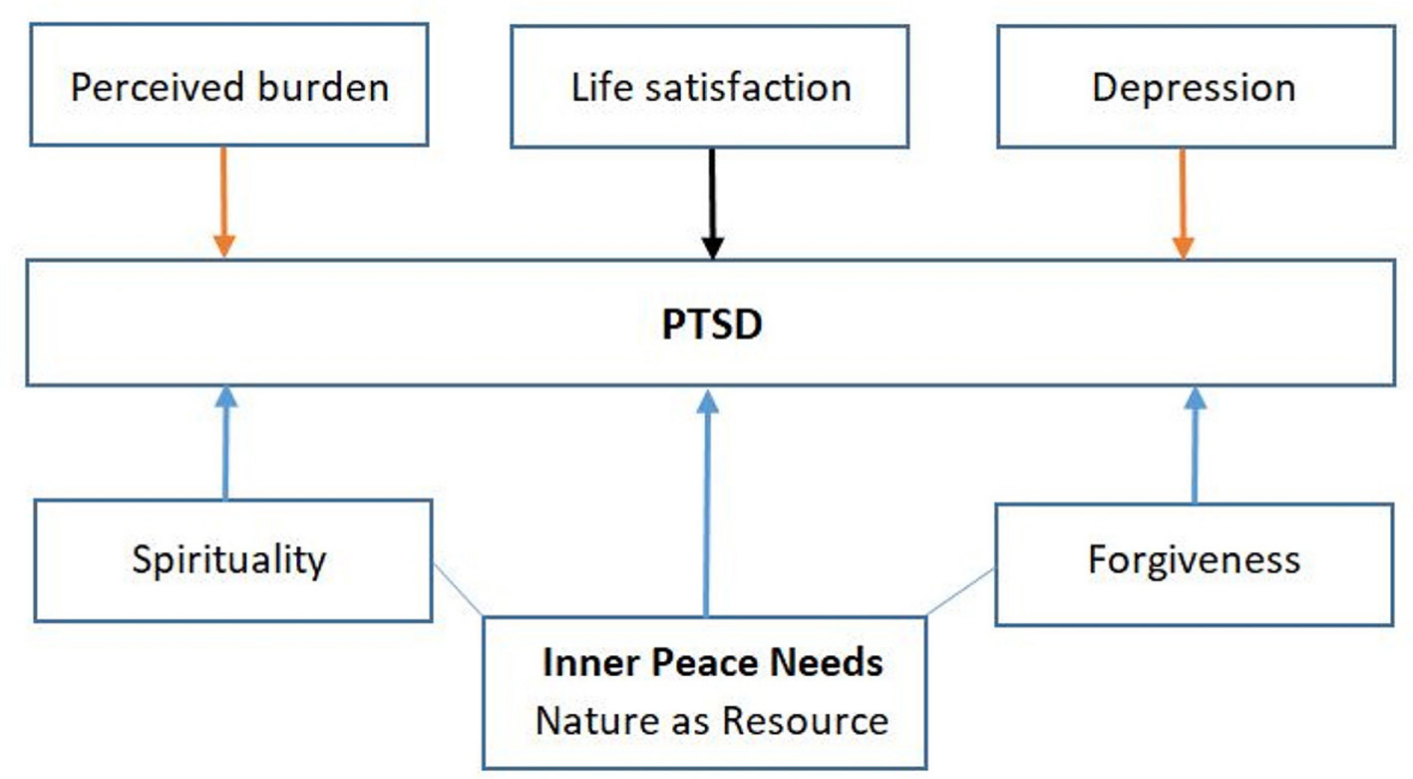
Model of supposed aggravating and buffering influences on patients' PTSD symptoms.

Thus, for this study, we focused on the following research questions: (1) To what extent do male war-traumatized patients in Croatia still feel a need for inner peace, particularly related to specific places of quietness and peace where the “beauty of nature” can be experienced? (2) Are these nature-related needs of psychiatrically treated war participants associated with forgiveness and religious trust? (3) Are inner peace needs and forgiveness needs predictors of patients' PTSD scores? The background rationale for these study questions is depicted as a model in Figure 1.

## 2. Materials and methods

### 2.1. Participants

Male participants were recruited in seven hospitals in Croatia, located in Popovaca (18%), Rijeka (13%), Nasice (7%), Strmac (6%), Vukovar (17%), Split (23%), and Zagreb (16%). These patients were treated because of post-traumatic stress disorder according to the International Classification of Diseases (ICD) (F43.1 according to ICD-10), an enduring personality change after a catastrophic experience (F62.0), or other mental health affections according to ICD-10 (i.e., depressive diseases and anxiety disorders). They were either inpatients, outpatients, or day clinic patients. Participants may have participated in the war actively as soldiers or “passively” as civilians.

Patients were informed about the purpose of the study, and they gave informed consent to anonymously participate in the cross-sectional survey. The study was approved by Freiburg University's ethical commission (votum 179/12) and consented to by the respective hospitals in Croatia.

### 2.2. Measures

Apart from basic sociodemographic data, participants responded to standardized measures in Croatian that are described in the following sections.

#### 2.2.1. Post-traumatic stress disorder symptoms (PCL-M)

Stressful war-related experiences in terms of PTSD were measured with the PTSD Checklist-Military Version (PCL-M) (38). The checklist addresses problems associated with psychological distress that soldiers and veterans may experience, such as repeated disturbing memories, thoughts, images, or dreams of a stressful military experience, physical reactions when reminded of a stressful military experience, and avoidance of activities or situations because they reminded participants of a stressful military experience (39, 40). We used a modified version with 17 items, which uses whole sentences instead of reduced sentences. The internal reliability of this 17-item version was very good in soldiers (Cronbach's alpha = 0.93) (37). The internal consistency was very good in this sample (Cronbach's alpha = 0.96).

The respective items were scored on a 5-point Likert scale ranging from 1 (not at all) to 5 (extremely). The total symptom severity score may range from 17 to 85. We did not use the checklist to diagnose PTSD but to screen individuals for perceived stressful experiences.

#### 2.2.2. Inner peace needs and forgiveness needs

From the Spiritual Needs Questionnaire (SpNQ) (41, 42), which differentiates Religious needs (Cronbach's alpha = 0.87 to 0.92), Existential (Reflection/Meaning) needs (Cronbach's alpha = 0.74 to 0.82), Inner Peace needs (Cronbach's alpha = 0.73 to 0.82), and Giving/Generativity needs (Cronbach's alpha =0.71 to 0.74), we used two specific subscales.

- The 3-item scale *Inner Peace needs* addresses the needs to “plunge into beauty of nature” (N6), to “dwell at a place of quietness and peace” (N7), and to “find inner peace” (N8). In this sample of male participants with psychiatric diseases, the scale has acceptable internal consistency (Cronbach's alpha = 0.64).
- The 5-item *Forgiveness*/*Clarification needs* scale (Cronbach's alpha = 0.71) (37) addresses the needs to “reflect back on your life” (N4), to “dissolve/clarify open aspects of your life” (N5), to “talk with others about your fears and worries” (N2), to “forgive someone from a distinct period of your life” (N16), and to “be forgiven” (N17). In this sample, the scale has good internal consistency (Cronbach's alpha = 0.76).

Participants rate how strong these needs were for them on a 4-point scale (0—not at all; 1—somewhat; 2—strong; and 3—very strong).

#### 2.2.3. Stress perception (PSS)

As stress contributes to PTSD symptoms and may trigger spiritual needs, we used Cohen's Perceived Stress Scale (PSS), which measures the perception of stress levels in specific situations during the last month (22). Internal reliability of the original PSS-10 was moderate (alpha = 0.78) (43). All items refer to emotions and thoughts and how often one may have felt or thought a certain way. The scores range from 1 (never) to 4 (very often); higher scores would thus indicate greater stress. The internal consistency of the scale in this sample is good (Cronbach's alpha = 0.74).

#### 2.2.4. Perceived burden (NRS)

Perceived impairment of daily life by the current life situation may trigger stress perception and PTSD symptoms. This perceived burden was addressed with a numeric rating scale (NRS) ranging from 0 (not at all) to 100 (unbearable). In this sample, Perceived Burden correlates strongly with stress perception (*r* = 0.51), low life satisfaction (*r* = 0.56), and moderately with depression (*r* = 0.43) and anxiety (*r* = 0.39).

#### 2.2.5. Anxiety and depression (HADS)

For symptoms of anxiety and depression, the Hospital Anxiety and Depression Scale (HADS) was used (44). It consists of 14 items using a 4-point Likert scale. The instrument measures the scales “anxiety” and “depression,” consisting of seven items each with a score range of 0–21. Higher scores indicate a greater likelihood of anxiety or depression. Items are, for example, “I feel tense or wound up,” “I still enjoy the thongs I used to,” “I feel as I am slowed down,” or “I look forward with enjoyment”. The internal consistency of the scale was reported to be good (44). In this sample, the internal consistency of the Anxiety subscale was good (Cronbach's alpha = 0.87) as well as for the Depression subscale (Cronbach's alpha = 0.84).

#### 2.2.6. Life satisfaction (BMLSS-10)

To measure overall life satisfaction, the Brief Multidimensional Life Satisfaction Scale (BMLSS) was used (45). This 10-item questionnaire addresses five main dimensions of life satisfaction: intrinsic (oneself and life in general), social (friendships and family life), external (work situation and habitation), prospective (financial situation and future prospects), and health (health situation and abilities to deal with daily life concerns). It can be scored from 0 (very unsatisfied) to 6 (very satisfied). The scale's internal consistency in this sample is very good (Cronbach's alpha = 0.93).

#### 2.2.7. Spiritual resources to cope (SpREUK)

As we assume that participants may have used their spirituality/faith as a resource to cope with their situation, we addressed this topic with the SpREUK questionnaire. The instrument was developed to investigate whether or not patients with chronic diseases rely on spirituality as a resource to cope with illness (46). It relies on essential motifs found in counseling interviews with chronic disease patients (i.e., having trust/faith; searching for a transcendent source to rely on; and reflecting on life and subsequent changes in life and behavior). In its 15-item version (SpREUK-15), it differentiates three factors (47):

- *Search (for Support*/*Access)* (Cronbach's alpha = 0.91) deals with patients' intention to find or have access to a spiritual/religious resource that may be beneficial to cope with illness and their interest in spiritual/religious issues (insight and renewed interest). In this sample, Cronbach's alpha is 0.92.
- *Trust (in Higher Guidance*/*Source)* (Cronbach's alpha = 0.91) is a measure of intrinsic religiosity dealing with patients' conviction to be connected with a higher source that carries through and to be sheltered and guided by this source—whatever may happen. In this sample, Cronbach's alpha is 0.90.
- *Reflection (Positive Interpretation of Disease)* (Cronbach's alpha = 0.86) deals with cognitive reappraisal because of illness and subsequent attempts to change (i.e., reflect on what is essential in life; hint to change life; chance for development; and illness has meaning). In this sample, Cronbach's alpha is 0.87.

The items were scored on a 5-point scale from disagreement to agreement [0—does not apply at all; 1—does not truly apply; 2—do not know (neither yes nor no); 3—applies quite a bit; 4—applies very much]. The scores can be referred to as a 100% level (transformed scale score).

Two items (f2.6: “To my mind, I am a religious individual” = R; f1.1: “To my mind, I am a spiritual individual” = S) were used to categorize participants as religious but not spiritual (R + S-), not religious but spiritual (R – S+), both religious and spiritual (R + S+), or neither religious nor spiritual (R – S–) (46).

### 2.3. Statistical analysis

We performed descriptive statistical analyses for the sociodemographic variables. Sub-sample analyses were reported using analysis of variance (ANOVA), assuming *p* <0.001 as significant. For better contextualization of results, effect sizes were described. With respect to these η^2^ values, scores <0.06 are considered small effects, between 0.06 and 0.14 moderate, and >0.14 strong.

For correlation analysis, we chose Spearman's rho coefficient since it is more robust with skewed data. Here, *r* <0.30 is considered small, between 0.30 and 0.50 moderate, and >0.50 strong.

Linear regression modeling with stepwise variable selection was used to evaluate predictors of Inner Peace needs and PTSD symptoms as dependent variables. Standardized beta coefficients are presented for model interpretation.

All statistical analyses were performed with SPSS 28.0.

## 3. Results

### 3.1. Description of the sample

The sample consists of 638 male patients (23% <45 years of age, 47% 45–55 years, and 30% >55 years) who were treated in seven psychiatric centers in Croatia. Among them, 68% were diagnosed with PTSD and 32% with other psychiatric diagnoses (Table 1).

**Table 1:** Description of the enrolled male patients from Croatia (n = 638)*.

	***N***	**%**	**Mean ±SD [range]**
Gender: male	638	100.00	
Age cohorts	598	100.0	
<45 years	138	23.2	
45–50 years	159	26.8	
51–55 years	117	19.7	
>55 years	180	30.3	
Educational level	634	100.0	
Primary school	114	18.0	
Middle school/Professional school	416	65.6	
High school/University	104	16.4	
Religious denomination	609	100.0	
Catholics	547	89.8	
Other	38	6.2	
Without	24	3.9	
Spiritual–religious self-categorization	578	100.0	
R+S+	250	43.3	
R + S–	233	40.0	
R – S+	55	9.5	
R – S–	40	6.9	
Active participation in the war	626	100.0	
Yes	496	79.2	
No	130	20.8	
Duration of participation in war	474	100.0	
<1 year	108	22.8	
1–3 years	83	17.5	
Whole period	283	59.7	
Treatment group	638	100.0	
PTSD	435	68.2	
Other disorders	203	31.8	
Inner Peace needs (SpNQ)	627		2.2 ± 0.7 [0–3]
Forgiveness/Clarification needs (SpNQ)	629		1.9 ± 0.7 [0–3]
Needs to forgive others	629		1.9 ± 1.0 [0–3]
Needs to be forgiven	629		1.7 ± 1.1 [0–3]
Stress perception (PSS)	636		26.1 ± 8.1 [5–48]
Perceived burden (NRS)	577		60.9 ± 26.4 [0–100]
PTSD (PCL-M)	524		56.7 ± 15.8 [2–85]
Anxiety (HADS)	625		16.5 ± 5.5 [2–28]
Depression (HADS)	625		16.5 ± 5.6 [6–28]
Life satisfaction (BMLSS)	625		51.1 ± 21.0 [0–100]
Search for access (SpREUK)	529		47.6 ± 31.5 [0–100]
Religious trust (SpREUK)	597		55.0 ± 31.0 [0–100]
Reflection: positive life construction (SpREUK)	590		49.6 ± 27.5 [0–100]

^*^Not all responded to all variables and thus the absolute number is sometimes lower than N = 638. The % refers to the number of responding persons (100%).

Among those without PTSD diagnoses, the following disorders were diagnosed: psychotic disorders (40%), depressive disorders (27%), mental and behavioral disorders caused by alcohol or opiates (12%), anxiety and obsessive-compulsive disorders (9%), and other (13%).

Most had actively participated in the Balkan war (79%), 60% of whom for the whole war period. 83% of active war participants were diagnosed with PTSD, as were 14% of those without active war participation. However, 60% of those with PTSD participated in the whole war period, and 40% of those with <3 years; the same proportions were found for those with other psychiatric diagnoses: 60% participated the whole war period, and 40% <3 years. Depending on the age cohorts, PTSD was diagnosed in 74–79% of men aged 45–50 years, 51–55 years, and >55 years, and these were the active war participants (83–94%). Among those <45 years of age, 49% were active war participants, and the others probably suffered from secondary war trauma. We do not know when they were diagnosed for the first time. However, among the active war participants, 26% have been in treatment for more than 20 years, 13% for 15–20 years, 23% for 10–15 years, 19% for 5–10 years, and 19% for 5 years. Among those who were not active war participants, 68% were treated for <11 years.

The majority of participants have a Catholic denomination (90%), and 83% regard themselves as religious (either R + S+ or R + S–).

Their general life satisfaction is in the mid-range (Table 1), indicating that they are definitely not satisfied with it. With a mean score of 61, their perceived burden is considerably high. Their PTSD scores and their stress perception are in the upper mid-range. With respect to the utilization of their spirituality as a resource to cope with their health situation or burden, the subscales of Search and Trust, as well as Reflection, scored in the indifferent mid-range (Table 1).

Their need to forgive others was strong in 34%, moderate in 34%, weak in 21%, and not perceived in 11%, while their need to be forgiven was strong in 28%, moderate in 31%, weak in 23%, and not perceived in 18%. The active forgiveness score was higher than the passive forgiveness score (Table 1).

All further data to describe the sample can be found in Table 1.

### 3.2. Intensity of nature and Inner Peace related needs

With respect to the Inner Peace needs subscale, we focused on three specific needs: to “plunge into the beauty of nature”, “dwell at a place of quietness and peace”, and “find inner peace”. As shown in Table 2, the strongest need was to dwell in a place of quietness and peace (66% expressed a strong need), followed by the need to find inner peace (57% expressed a strong need), and third to “plunge in the beauty of nature” (47% expressed a strong need). Only a few had none of these needs (<10%).

**Table 2:** Intensity of nature and Inner Peace related needs.

		**Intensity of needs (%)**				
**Item ID**	**During the last time, did you have had the needs to …**	**Not**	**Somewhat**	**Strong**	**Very strong**	**Mean score**
N6	Plunge into beauty of nature?	10.1	16.8	26.4	46.7	2.1 ± 1.0 [0–3]
N7	Dwell at a place of quietness and peace?	4.5	8.4	21.6	65.5	2.5 ± 0.8 [0–3]
N8	Find inner peace?	7.6	12.7	22.4	57.2	2.3 ± 1.0 [0–3]

For the following analyses, we will use both the three specific items and the scores of the Inner Peace needs scale. As shown in Table 3, age was of low relevance for these nature and inner peace-related needs, while spiritual/religious self-categorization had an influence on the need to plunge into the beauty of nature and to find inner peace, albeit with a small effect size. Patients treated for PTSD had significantly higher needs to dwell in a place of quietness and peace and to find inner peace as compared to patients with other psychiatric diagnoses, resulting in a significantly higher score on the Inner Peace needs scale.

**Table 3:** Intensity of nature and Inner Peace needs in subgroups (ANOVA).

		**N6 plunge into beauty of nature**	**N7 dwell at place of quietness and peace**	**N8 find Inner Peace**	**Inner Peace needs (SpNQ)**
**All ages**	Mean	2.10	2.50	2.33	2.19
	SD	1.01	0.82	0.94	0.67
<45 years	Mean	2.13	2.40	2.43	2.17
	SD	1.01	0.85	0.89	0.70
45–50 years	Mean	2.01	2.52	2.24	2.14
	SD	1.04	0.82	0.98	0.68
51–55 years	Mean	1.89	2.50	2.25	2.12
	SD	1.11	0.86	0.98	0.72
>55 years	Mean	2.31	2.55	2.37	2.30
	SD	0.87	0.79	0.91	0.61
*F* value		4.75	0.96	1.32	1.04
η^2^ value		0.02	0.01	0.01	0.01
*p*-value		**0.003**	n.s.	n.s.	n.s.
**All SpR responder**	Mean	2.08	2.50	2.33	2.19
	SD	1.03	0.83	0.93	0.67
R + S+	Mean	2.27	2.61	2.54	2.35
	SD	0.96	0.75	0.79	0.60
R + S–	Mean	1.91	2.41	2.16	2.05
	SD	1.05	0.88	1.02	0.71
R – S+	Mean	1.96	2.52	2.08	2.09
	SD	1.13	0.82	1.05	0.63
R – S–	Mean	2.05	2.30	2.32	2.15
	SD	0.92	0.85	0.80	0.61
F value		5.21	3.22	8.27	9.36
η^2^ value		**0.03**	0.02	**0.04**	**0.05**
*p*-value		**0.001**	0.022	**<0.0001**	**<0.0001**
**All treatment groups**	Mean	2.10	2.48	2.29	2.17
	SD	1.02	0.83	0.96	0.68
PTSD treatment	Mean	2.07	2.60	2.37	2.24
	SD	1.03	0.75	0.92	0.62
Other disorders	Mean	2.15	2.22	2.14	2.03
	SD	0.98	0.93	1.02	0.77
*F* value		0.87	29.89	7.71	12.72
η^2^ value		0.00	**0.05**	0.01	0.02
*p*-value		n.s.	**<0.0001**	**0.006**	**<0.0001**

Bold values: noteworthy η^2^ values and significant *p*-values. n.s.: not significant.

Those who had been actively participating in the war, as a trend, had higher needs to dwell in a place of quietness and peace (*F* = 5.9, *p* = 0.016; η^2^ = 0.01). Interestingly, those patients who had been at war for <1 year had the highest need to find inner peace as compared to those who had participated longer (*F* = 4.9, *p* = 0.008; η^2^ = 0.02), while their other needs did not differ significantly (data not shown). Religious denominations or patients' educational levels had no significant influence on these three needs or the score of the Inner Peace needs scale (data not shown).

### 3.3. Correlations between nature and Inner Peace needs and indicators of mental health affections

The need to dwell in a place of quietness and peace was much strongly related to the need to finding inner peace than the need to “plunge into the beauty of nature” (Table 4). Nevertheless, this nature-related need is moderately associated with the need to find such places to relax and recover. Among these specific needs, finding inner peace was strongest and moderately related to Forgiveness/Clarification needs.

**Table 4:** Correlation analyses.

	**N6 plunge into beauty of nature**	**N7 dwell at place of quietness and peace**	**N8 find Inner Peace**	**Inner Peace needs (SpNQ)**
N6 plunge into beauty of nature	1.000			
N7 dwell at place of quietness and peace	0.321^**^	1.000		
N8 find inner peace	0.265^**^	0.497^**^	1.000	
Inner Peace needs (SpNQ)	0.671^**^	0.654^**^	0.703^**^	1.000
Forgiveness/Clarification needs (SpNQ)	0.297^**^	0.303^**^	0.447^**^	0.651^**^
**Quality of life indicators**				
Stress perception (PSS)	−0.020	0.245^**^	0.229^**^	0.241^**^
Perception of burden (NRS)	−0.013	0.304^**^	0.258^**^	0.343^**^
PTSD (PCL-M)	−0.133^**^	0.292^**^	0.224^**^	0.220^**^
Anxiety (HADS)	−0.054	0.181^**^	0.115^**^	0.154^**^
Depression (HADS)	−0.113^**^	0.211^**^	0.153^**^	0.137^**^
Life satisfaction (BMLSS)	0.173^**^	−0.220^**^	−0.176^**^	−0.143^**^
**Spirituality as a resource**				
Search for access (SpREUK)	0.172^**^	0.165^**^	0.217^**^	0.305^**^
Religious trust (SpREUK)	0.175^**^	0.165^**^	0.169^**^	0.222^**^
Reflection: positive life construction (SpREUK)	0.209^**^	0.070	0.116^**^	0.204^**^

^**^p <0.0001 (Spearman rho); weak (light yellow), moderate (yellow), and strong (orange) correlations are highlighted.

As the needs to find places to dwell in quietness and peace and thus to find inner peace were clearly interconnected, both were best related to the perception of burden and stress and PTSD symptoms, while the need to “plunge into the beauty of nature” was marginally or not at all related (Table 4). In addition, depression and low life satisfaction were only weakly related to the need to dwell in places of quietness and peace and not relevantly related to the other two needs.

Patients' Search for access to a spiritual resource that may help them cope with their situation was moderately related to the Inner Peace needs scale, while religious Trust and Reflection were related to this factor only weakly (Table 4). Finally, Forgiveness/Clarification needs were moderately related to SpREUK's Search scale (*r* = 0.37) and Reflection (*r* = 0.34) and weakly related only to religious Trust (*r* = 0.25) (data not shown).

### 3.4. Predictors of Inner Peace needs

As there were several variables significantly related to patients' Inner Peace needs (SpNQ), we performed stepwise regression analyses and included significantly related independent variables to clarify which of these would predict Inner Peace needs as a dependent variable best (Table 5).

**Table 5:** Regression analyses with Inner Peace needs (SpNQ) as the dependent variable.

**Dependent variable: Inner Peace needs (SpNQ) Model 3: *F* = 124.2, *p* <0.001; *R*^2^ = 0.49**	**Beta**	***T***	***p***
(constant)		1.686	0.093
Forgiveness/Clarification needs (SpNQ)	0.640	17.032	<0.001
PTSD (PCL-M)	0.217	4.449	<0.001
Life satisfaction (BMLSS-10)	0.120	2.494	0.013

Excluded from the model: duration of war participation, Stress perception (PSS), Perceived burden (NRS), Anxiety (HADS), Depression (HADS), Search (SpREUK), and Religious Trust (SpREUK).

Here, Forgiveness/Clarification needs were the best predictors of patients' Inner Peace needs (explaining 47% of the variance), followed by PTSD symptoms (adding 2% of the further explained variance) and life satisfaction (adding 1%). These three variables would together predict 49% of the variance. Duration of war participation, stress perception and perceived burden, anxiety and depression, and search and religious trust had no independent influence in this regression model.

### 3.5. Predictors of PTSD symptoms

As we aimed to analyze which variables may buffer patients' PTSD symptoms, we again performed stepwise regression analyses and included the theoretically related variables as independent variables.

As shown in Table 6, patients' life satisfaction was the best predictor (explaining alone 41% of the variance), followed by perceived burden (adding 11%) and depressive symptoms (adding 4%), and further, Inner Peace needs (adding 2%), religious Trust (adding 1%), and duration of war participation (adding 0.5% of explained variance). These six variables together would predict 60% of PTSD variance. Stress perception, Anxiety, Search, and Forgiveness/Clarification needs had no independent influence in this regression model.

**Table 6:** Regression analyses with PTSD symptoms (PCL-M) as the dependent variable.

**Dependent variable: PTSD (PCL-M) Model 6: *F* = 95.4, *p* <0.001; R^2^ = 0.60**	**Beta**	***T***	***p***
(constant)		11.39	<0.001
Life satisfaction (BMLSS-10)	−0.38	−8.81	<0.001
Perceived burden (NRS)	0.30	7.12	<0.001
Depressive symptoms (HADS)	0.24	6.30	<0.001
Inner Peace needs (SpNQ)	0.14	3.88	<0.001
Religious trust (SpREUK)	0.11	3.25	0.001
Duration of war participation	0.075	2.28	0.023

Excluded from the model: Stress perception (PSS), Anxiety (HADS), Search (SpREUK), and Forgiveness/Clarification needs (SpNQ).

## 4. Discussion

Even more than 25 years after the end of the Balkan War, male war participants in Croatia are in psychiatric treatment because of their war experiences. Their need to dwell in places of quietness and peace, to find inner peace, and to merge into the beauty of nature scored high. These needs to find inner peace may be considered desires to escape from their emotional pain and disturbing memories. Their need for specific places of quietness and peace in the external world and thereby finding inner peace could be interpreted as a metaphor for their longing for wholeness, integrity, and safety in contrast to the ongoing impact of unresolved issues.

However, these Inner Peace needs were only marginally related to anxiety and depression, weakly related to stress perception or PTSD symptoms, and strongly related to Forgiveness/Clarification needs. These latter needs to clarify open aspects of their lives and to forgive and to be forgiven (Forgiveness/Clarification needs) were the best predictors of their Inner Peace needs. These results underline that the patients from Croatia still have to struggle with their experiences, moral injuries, physical and mental trauma, and guilt. It is not post-traumatic stress alone, however, that triggers patients' Inner Peace needs, but their inability to cope with the burden and their unresolved problems dating back to the time of war. Reflecting on life means (among other aspects), to consider unresolved problems, situations of hurt and shame, may thus result in needs of forgiveness and to be forgiven. In German soldiers who returned from international missions, the best predictors of these Forgiveness/Clarification needs were PTSD symptoms and stress perception (37), which is not the case, however, in the Croatian veteran sample.

Among German soldiers, these reflexive needs were strongly related to Inner Peace needs. A similar correlation is present in this sample of male patients from Croatia, too. A meta-analysis confirms positive correlations between forgiveness and mental health (48). Moreover, those who report unforgiveness by others can nevertheless buffer depression when one is able to forgive oneself (49). Although men are most likely to be ready to forgive others, their perception of unforgiveness by others is related to their depressive symptoms (49). A study by Ingersoll-Dayton et al. (50) confirmed that unforgiveness by others has a direct effect on depressive symptoms and an additional indirect effect that works through self-forgiveness and rumination. This underlines the relevance of being forgiven, forgiving oneself, and forgiving others. Obviously, this requires a complex and probably long process of reconciliation. In this study of male patients from Croatia, the need to forgive others was higher than the need to be forgiven; these needs were expressed strongly by 34% and 28%, respectively. In other words, there is still a noteworthy burden affecting Croatian post-war patients. Training programs addressing forgiveness might be an interesting option for them, as was shown by the REACH Forgiveness psychoeducation program (51). In that study, when the offender was of similar spirituality or faith, the program was more effective (51). Transferred to the situation of Croatian patients, there are no general reconciliation programs between Croatian victims (who are mainly Catholics) and Serbian aggressors (who are mainly Orthodox Christians); both groups being Christians may facilitate forgiving the former aggressor and coping with their mental injuries; this would presuppose, however, that they overcome nationalist identifications of religious denomination and nationality. The role of the respective churches and their spiritual and pastoral care in this could be crucial to facilitating reconciliatory processes on individual, group, and national levels.

When it is true that a person's faith and belief in God may have an indirect positive effect on the link between forgiveness and mental health (50), then one could expect a stabilizing effect from this resource. However, Forgiveness/Clarification needs were moderately related to Search (for access to a supportive spiritual resource) and Reflection of life concerns and weakly related to religious Trust (although most participants in this study were Catholics by denomination). Thus, it is foremost patients' search for an (unknown) supportive spiritual source (providing “Inner Peace”) rather than having trust in God, which relates to the intention to clarify the burdening things in life and to forgive.

Interestingly, religious Trust and Inner Peace needs were among the positive predictors of PTSD symptoms. This means the higher the PTSD symptoms are, the higher patients score on religious Trust and the more they try to find inner peace. Patients' faith was thus utilized as a resource to cope with the burden, which further triggers their need to find inner peace. As one may expect, the best predictors of PTSD symptoms were low life satisfaction, perceived burden in life, and depressive symptoms.

The needs for places of quietness and peace were the strongest Inner Peace needs in the patients of our sample. Such places, e.g., in nature, are probably intended to distance themselves from their burden in terms of an escape strategy. Maybe these places to find inner peace are more of a desire than a reality. Nevertheless, experiencing nature (either green or blue spaces) is recognized to improve psychological wellbeing (33, 34) and reduce stress (35). Yet, the accessibility of green spaces is one of the crucial points; nevertheless, their availability does not guarantee positive effects on mental health (52). A systematic review of longitudinal observational studies on exposure to green and blue spaces in correlation with mental health revealed protective effects in some studies but underlined that the associations are weak and that the evidence is less than consistent (53). The fact is, however, that the traumatized patients from Croatia strongly long for such and similar places of quietness, hoping they may find inner peace. It seems that by longing for special or extraordinary places of quietness and peace, e.g., in nature, male Croatian veterans regard these as a potential source to distance themselves from their emotional pain and burden in order to find (more) inner peace and reconciliation. Whether this expectation is true remains to be seen. Again, spiritual and pastoral care by the religious communities could find indications in these findings to set up facilitating strategies for peace and reconciliation, for individuals, groups, and maybe even populations formerly in conflict and war.

### 4.1. Limitations

Due to the cross-sectional design of the study, no causal conclusions can be drawn. The participants were treated because of their post-war burden, even more than 25 years after the Balkan War. However, we have no data on the general population in Croatia in terms of their Inner Peace needs or their Clarification/Forgiveness needs.

In this study, we did not directly compare the data of Croatian patients and patients from Bosnia and Herzegovina. Comparative data of Muslim patients from Sarajevo (Bosnia Herzegovina) show that civilians from Sarajevo have significantly higher Inner Peace needs than Balkan War soldiers from Sarajevo (54). These needs were slightly higher than the Inner Peace needs in the Croatian sample. Their forgiveness needs have not yet been evaluated.

All patients were diagnosed and treated at the respective hospitals. Thus, we did not perform further diagnostic interviews or further analysis based on comorbidities within the PTSD sample.

### 4.2. Conclusion

Male post-war victims in Croatia have high Inner Peace needs. They indicate their intention to finally get rid of their disturbing experiences and to find inner peace. They hope to find it, particularly at specific places of quietness and peacefulness. While we assume that such places could be seen as a metaphor for longing for wholeness, integrity, and safety in contrast to the ongoing impact of unresolved issues, such places could nevertheless facilitate processes of reconciliation—still in the absence of their former enemies. Apart from, or in addition to, psychotherapeutic treatment, sheltered, peaceful places of inspiration and reconciliation might be an element to improve the difficult situation of long-term post-war patients still suffering from their war experiences.

## Data availability statement

The raw data supporting the conclusions of this article will be made available by the authors, without undue reservation.

## Ethics statement

The study was approved by the Freiburg University's Ethical Commission (votum 179/12) and consented by the respective hospitals in Croatia. The studies were conducted in accordance with the Helsinki declaration, the local legislation and institutional requirement. Written informed consent for participation in this study was provided by the participants' legal guardians/next of kin.

## Author contributions

AG and KB designed the study. AG established the research contacts and questionnaire distribution. AB and AG analyzed the data. All authors contributed to interpreting the data in writing and consented to the manuscript.
